# Supplementary material for: Frequency conversion of highly charged femtosecond spatiotemporal optical vortices in cascaded nonlinear crystals
Source: Nat Commun. 2026 Jul 29;17:9180. doi: 10.1038/s41467-026-75464-0 (PMC13521892; doi:10.1038/s41467-026-75464-0)
Supplement: Supplementary file 1 [file 41467_2026_75464_MOESM1_ESM.pdf]

Supplementary information: Frequency conversion of highly charged femtosecond spatiotemporal optical vortices in cascaded nonlinear crystals

Qingqing Liang,[1] Dan Wang,[1] Qiyuan Zhang,[1] Jianhua Hu,[1] Enliang Zhang,[1] Ming Wang,[1] Jinxin Wu,[1] Grover A. Swartzlander,[2] Yi Liu,[1,*]

[1]Shanghai Key Lab of Modern Optical System, University of Shanghai for Science and Technology, 516, Jungong Road, 200093 Shanghai, China

[2]Chester F. Carlson Center for Imaging Science, Rochester Institute of Technology, Rochester, New York, USA

[*] Corresponding author: yi.liu@usst.edu.cn.

Section 1: Theoretical Validation and Experimental Benchmarking of STOV Generation

We confirmed our STOV generating approach through simulations and experiments. Employing the space-time coupling framework [1,2], we calculated diffraction patterns for STOV fields with topological charges from $l^{(\omega)} = 0$ to 14 (Supplementary Fig. 1). These simulations incorporated actual experimental parameters, including the 35-fs pulse duration and 4 mm beam width. Experimentally, our 4f pulse shaper using SLM produced well-defined 800 nm STOV fields. Their diffraction patterns consistently produced black fringes (Fig. 2), matching our calculations completely across all topological charges. Spectral measurements found important differences: conventional Gaussian pulses ($l^{(\omega)}=0$) maintained position-independent spectra centered at 800 nm (Supplementary Fig. 2a). In contrast, STOV fields ($l^{(\omega)}=4$) exhibited distinct spatial-spectral chirp across the beam profile (Fig. 3a). This pattern coincides with theoretical expectations of spatiotemporal phase gradients [2], demonstrating both our technique's reliability and the intrinsic space-time coupling in STOV fields.

The ultimate fidelity and maximum achievable topological charge (ℓ) of the generated STOVs are constrained by the spatial resolution (i.e., pixel pitch) of the wavefront-shaping device. For the fundamental pulse, a very high ℓ requires an extremely steep azimuthal phase ramp. When this ramp is discretized by the finite pixels of the spatial light modulator (SLM), sampling errors occur, leading to a distorted phase structure in the central region of the STOV. Upon diffraction, this distortion manifests as the intensified central spot observed in the far-field patterns at the highest charges (e.g., $\ell^{(2\omega)} = 60$, Fig. 4b). Light from the distorted core contributes a diffuse background that partially fills the central null of the ideal vortex. It is important to note that the second-harmonic process itself imposes no fundamental restriction on ℓ, which enabled the high charges in our work. In contrast, the subsequent third-harmonic generation is more susceptible to degradation due to spatiotemporal walk-off between the interacting pulses. Future implementations employing SLMs with a finer pixel pitch could mitigate these discretization artifacts, thereby pushing the attainable topological charge even higher.

Section 2: Nonlinear Crystal Thickness-Dependent Topological Charge Conservation

In this section, we explored how BBO crystal thickness impacts topological charge preservation during harmonic conversion. For second-harmonic generation (SHG) with

0.3-mm-thick crystals, diffraction patterns preserved crisp features with excellent contrast (γ = 0.92 ± 0.03 at $l^{(2\omega)}$=20), demonstrating good topological charge conservation. However, thicker 1.0-mm crystals dramatically reduced contrast (γ=0.61 ± 0.05). We explain this blurring (Supplementary Fig. 3) to spatiotemporal astigmatism produced by group velocity mismatch ($\Delta v_g = v_g^{(800)} - v_g^{(400)}$ =0.87 mm/ps), where temporal walk-off affects the harmonic field's phase structure. Third-harmonic generation demonstrated greater sensitivity to crystal thickness. Based on the dispersion properties of Type-I BBO, the group velocity mismatch between 800 nm and 400 nm is approximately $\Delta(1/v_g) \approx 194$ fs/mm [3]. Therefore, for a 1 mm thick crystal, the temporal walk-off is $\Delta t = L \times \Delta(1/v_g) \approx 194$ fs. With 1-mm crystals, the 194-fs temporal walk-off showed substantial pattern degradation for $l^{(3\omega)} \geq 15$ (Supplementary Fig. 4). Detailed research indicated these fuzzy patterns relate to mixed topological charges ($l^{(3\omega)} = 3l^{(\omega)} \pm \delta$) due to partial decoherence between fundamental and harmonic fields. These findings show crystals should meet $d_{max} < \tau_{FWHM} /(2\Delta v_g)$ to sustain topological integrity.

In addition to this temporal walk-off, spatial walk-off also plays a role. In type-I BBO, the walk-off angle between the 800 nm and 400 nm beams is about 4.28° [3]. This leads to a spatial separation at the crystal exit face approximately 75 μm for a 1-mm-thick crystal. While this separation is negligible compared to our beam diameter (~4.0 mm) for the SHG process, it becomes significant for the subsequent SFG stage. The 800 nm and 400 nm beams propagate freely over ~20 mm between the two crystals. Due to the walk-off angle, this free propagation results in a transverse separation of ~1.48 mm when the beams reach the SFG crystal, substantially reducing their spatial overlap and thus degrading SFG efficiency. This major separation arises primarily from the free propagation between crystals, not from propagation within the SHG crystal. The thickness of the SHG crystal itself does not significantly alter the spatial walk-off effect relevant to the SFG process.

Section 3: Spectral-Temporal Control of Harmonic STOV Wavelengths

We tuned harmonic wavelengths by altering the spectral position of the phase singularity. Shifting the vortex center throughout the basic spectrum (786–819 nm in ~2.5 nm steps) while optimizing the BBO angle (~0.2°/nm offset) regulated phase matching. As seen in Supplementary Fig. 5a, this asymmetrically transformed fundamental STOV diffraction patterns while keeping high-quality second harmonics (Supplementary Fig. 5b). The harmonic wavelength shifted linearly with the singularity position ($\Delta\lambda^{(2\omega)} \approx 0.5\,\Delta\lambda^{(\omega)}$),

covering 393–409.5 nm (Supplementary Fig. 5c) without impacting topological charge conservation ($l^{(2\omega)} = 2l^{(\omega)}$) or diffraction clarity. This approach was further extended to third-harmonic generation via sum-frequency generation. By optimizing a second BBO crystal (type I, θ = 43.0°), we obtained tunable ultraviolet STOVs (263–274 nm; Supplementary Fig. 5d, e) that likewise obeyed topological charge conservation ($l^{(3\omega)} = 3l^{(\omega)}$). This tunability—achieved through coordinated spectral-phase control and nonlinear optics—enables wavelength-specific UV STOV production for applications like OAM communications and ultrafast spectroscopy.

Section 4: Phase-Matching Bandwidth Calculations for BBO Crystals

The acceptance bandwidth for critically phase-matched processes in birefringent crystals is primarily limited by group velocity mismatch (GVM). For a crystal of length L, the FWHM spectral bandwidth (in wavelength units) can be estimated as:

$$\Delta\lambda \approx 0.443 * \lambda / (L * |\partial(\Delta k)/\partial\lambda|) \qquad \text{(S1)}$$

where λ is the wavelength of interest (typically the fundamental wavelength for SHG), and $\Delta\boldsymbol{k} = \boldsymbol{k}_2 - 2\boldsymbol{k}_1$ (for SHG) or $\Delta\boldsymbol{k} = \boldsymbol{k}_3 - \boldsymbol{k}_1 - \boldsymbol{k}_2$ (for SFG) is the wavevector mismatch. The derivative $\partial(\Delta k)/\partial\lambda$ is calculated using the refractive indices and their dispersions, derived from the Sellmeier equations for BBO [3].

For our experiment (Type-I, θ = 29.2°, L = 100 μm), we have $|\partial(\Delta k)/\partial\lambda|_{800} \approx 8.86 \times 10^4$ m^{-1} μm^{-1}. Inserting into Eq. (S1) with λ = 800 nm yields $\Delta\lambda_{SHG}$ (800 nm) ≈ 50 nm (FWHM). This broad bandwidth confirms that the entire spectrum of our shaped fundamental pulse can participate in the SHG process, supporting the wide tunability observed. For the SFG stage (Type-I, θ = 43.0°, L = 100 μm), the bandwidth is constrained by the GVM for both input beams. We calculate the acceptance bandwidths separately. For the 800 nm beam: $|\partial(\Delta k)/\partial\lambda_{800}| \approx 5.54 \times 10^4$ m^{-1} μm^{-1} → $\Delta\lambda_{SFG}$ (800 nm) ≈ 12.5 nm. For the 400 nm beam: $|\partial(\Delta k)/\partial\lambda_{400}| \approx 2.95 \times 10^5$ m^{-1} μm^{-1} → $\Delta\lambda_{SFG}$ (400 nm) ≈ 5.5 nm. The narrower bandwidth, especially for the 400 nm input, dictates the spectral selectivity of the SFG process and is a key factor influencing the overall third-harmonic conversion efficiency.

The disparity between these bandwidths and our fundamental laser bandwidth (~30 nm) has direct consequences. In SHG, the large $\Delta\lambda_{SHG}$ enables robust wavelength tuning of the second-harmonic STOV simply by shifting the spectral singularity. In SFG, the small $\Delta\lambda_{SFG}$ (400 nm) means only a fraction of the second-harmonic spectrum contributes efficiently to third-harmonic generation. This necessitates careful alignment and partially explains the

lower efficiency and greater sensitivity to crystal thickness observed for the 266 nm STOV generation.

Figures

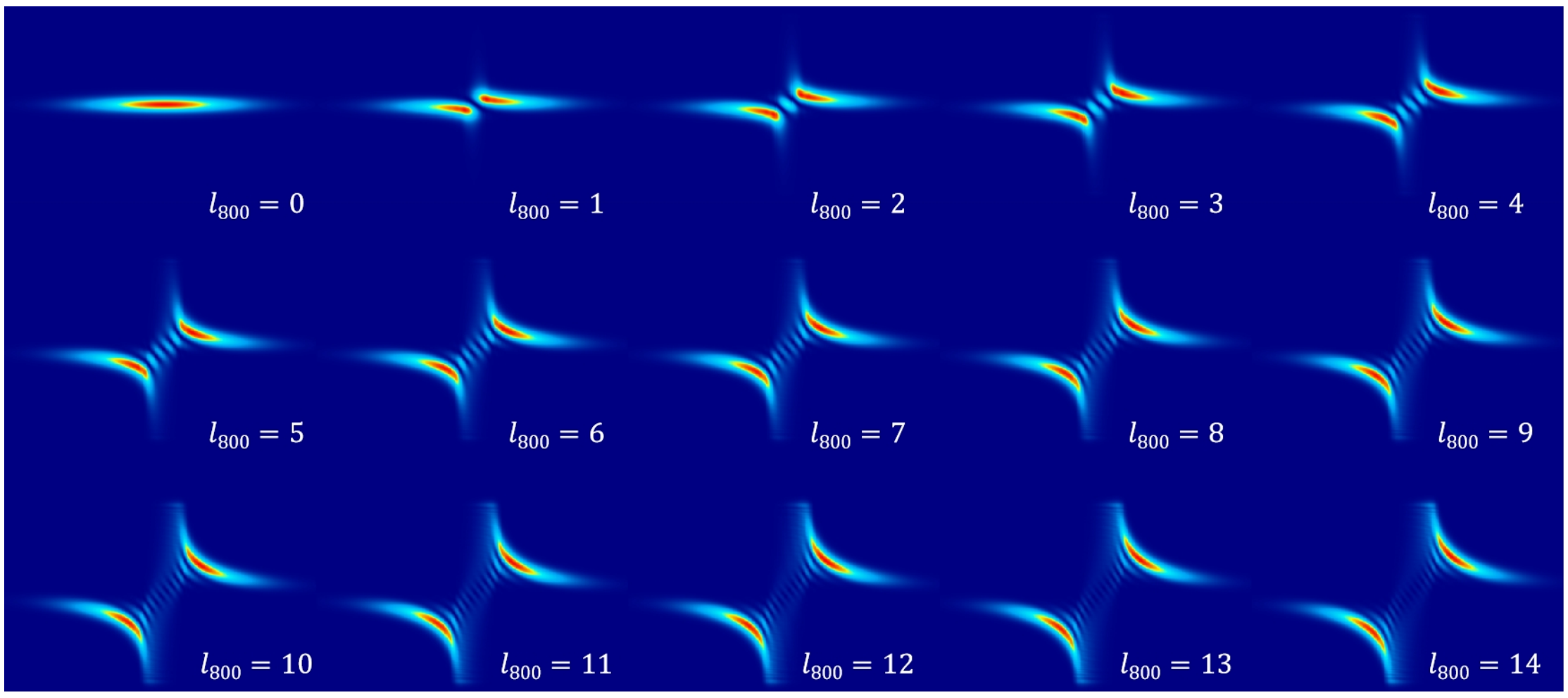

Supplementary Fig. 1. Calculated diffraction patterns of the 800 nm STOV field with different topological charges. With the calculated parameters chosen to those of the experiments.

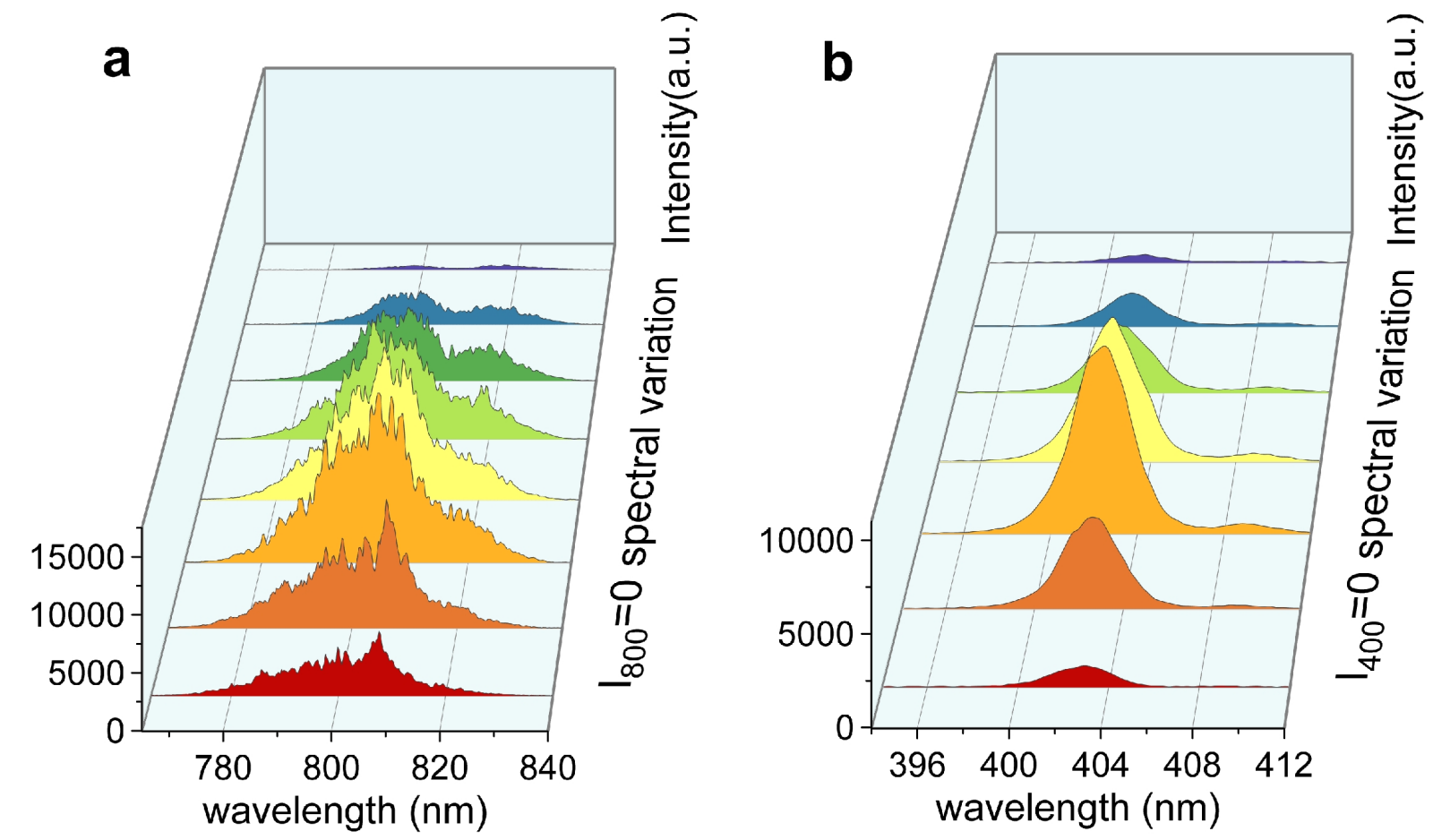

Supplementary Fig. 2. Spectrum measurement across the transverse direction of the 800 nm and 400 nm beam without topological charge. a, results for 800 nm field with $l^{(\omega)} = 0$. **b**, results for 400 nm field with $l^{(2\omega)} = 0$.

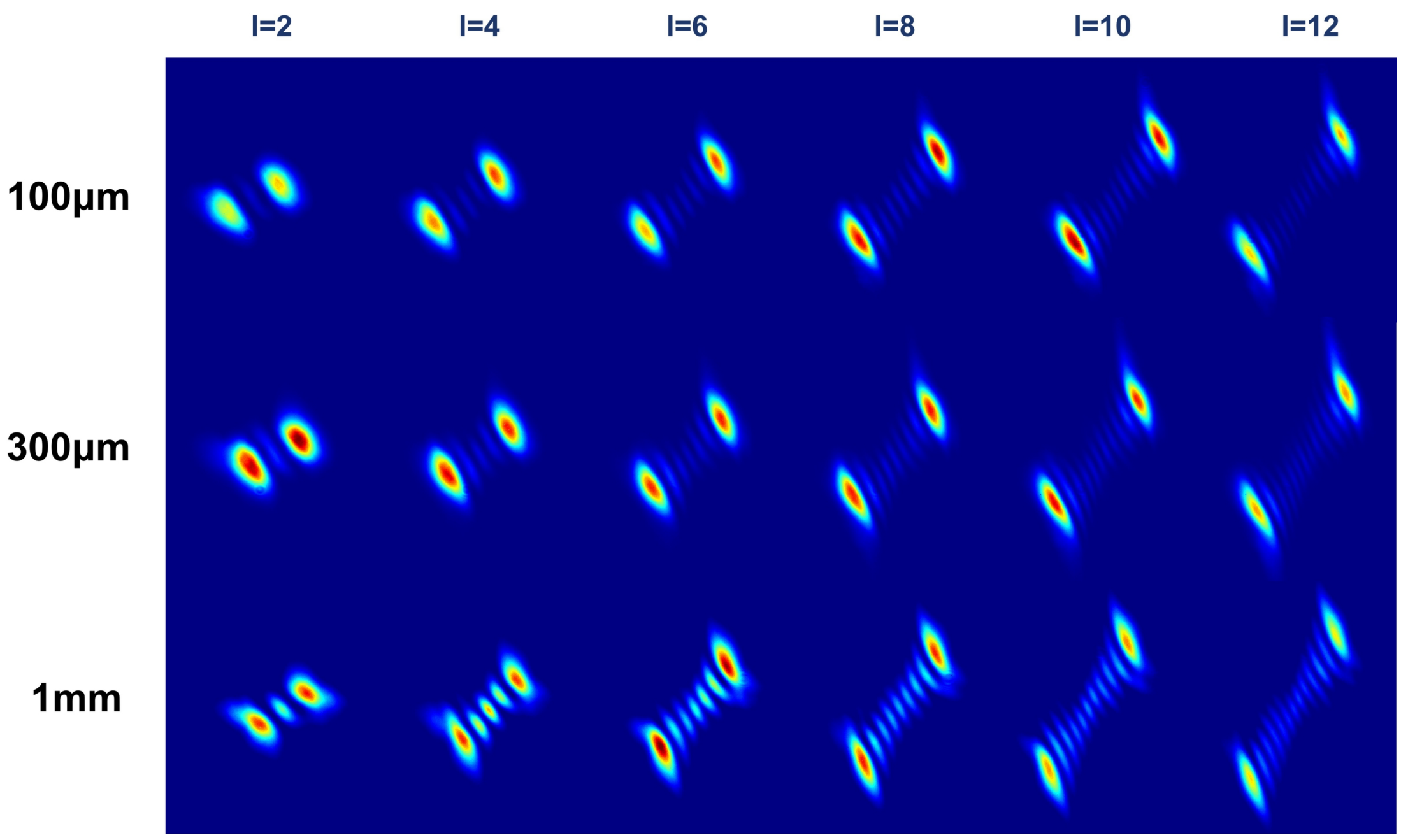

Supplementary Fig. 3. Diffraction patterns of the STOV field for different thickness of SHG crystals. The topological charge and the thickness of the crystals are denoted in the figure. With crystal of 1 mm, the diffraction patterns become blurred due to the spatiotemporal astigmatism.

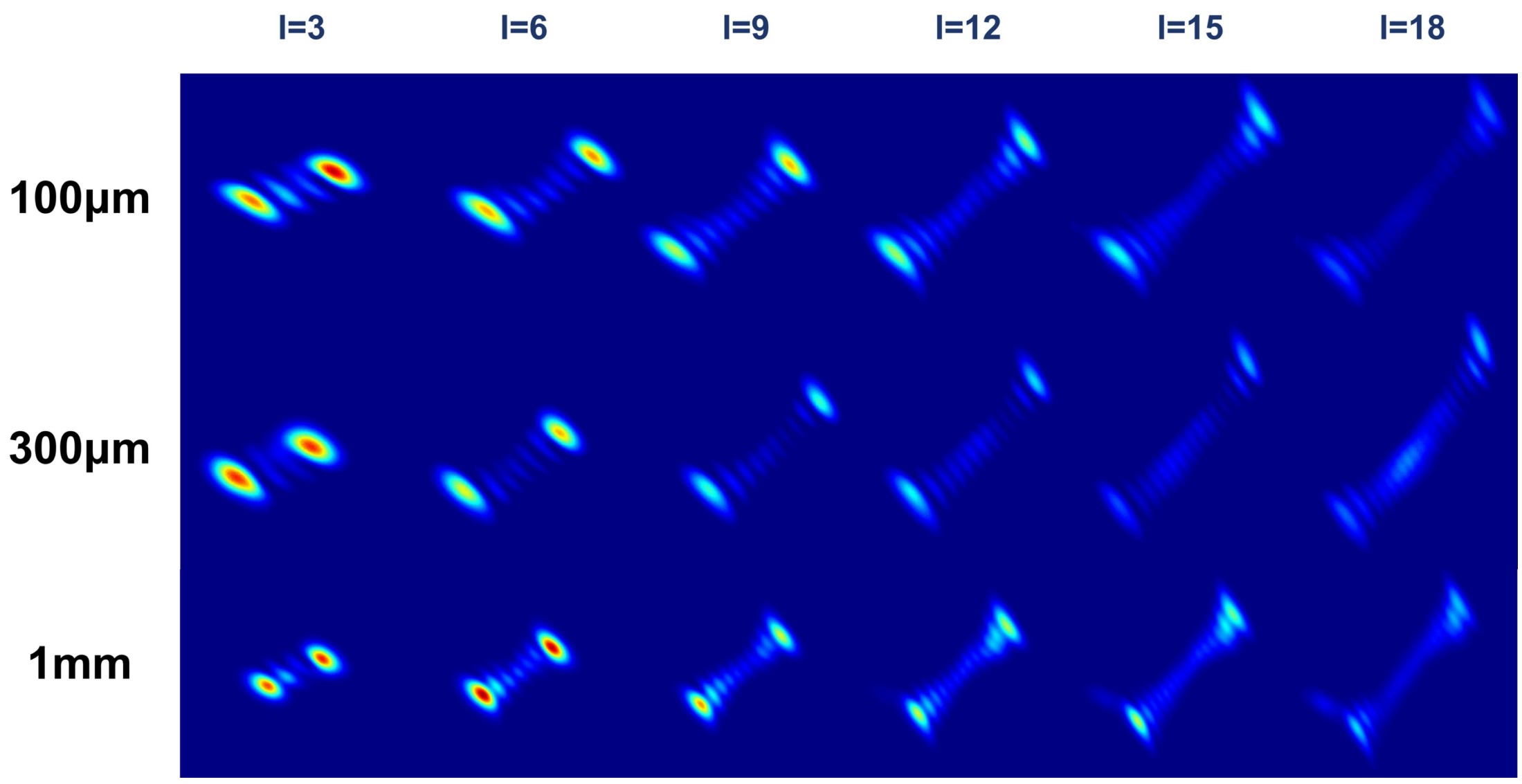

Supplementary Fig. 4. Diffraction patterns of the third harmonic STOV field at 266 nm for different thickness of SFG crystals. The topological charge and the thickness of the crystals are denoted. For higher topological charges and with SFG crystal of 1 mm, the diffraction patterns become blurred due to the spatiotemporal astigmatism and temporal walkoff inside the SFG crystal.

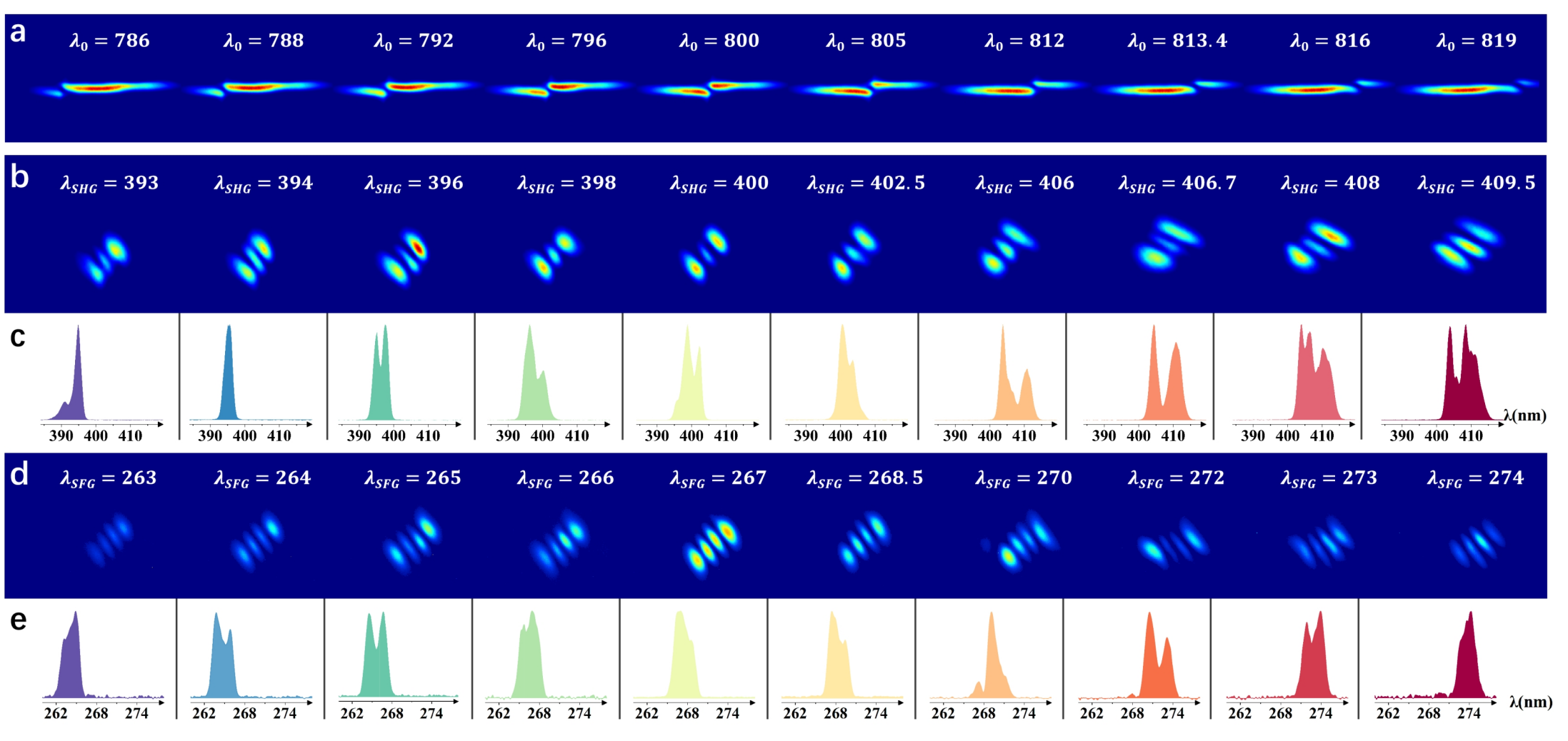

Supplementary Fig. 5. Tunability of the second and third harmonic STOV field. a: the diffraction patterns of the 800 nm STOV field with the topological charge located at varying wavelength, with the corresponding wavelength indicated in the tables above. b and c: the corresponding 400 nm STOV field and the measured spectrum of the second harmonic STOV field, with its central wavelength tuned from 393 nm to 409.5 nm. d and e: the corresponding 266 nm STOV field and the measured spectrum of the third harmonic STOV field, with its central wavelength tuned from 263 nm to 274 nm. The numbers above the figure present the central wavelength where the phase singularity was applied on the SLM and the central wavelength of the harmonic STOV.

References :

1. Hancock, S. W., Zahedpour, S., Goffin, A. & Milchberg, H. M. Free-space propagation of spatiotemporal optical vortices. *Optica* **6**, 1547 (2019).
2. Huang, S., Wang, P., Shen, X., Liu, J. & Li, R. Diffraction properties of light with transverse orbital angular momentum. *Optica* **9**, 469 (2022).
3. Dmitriev, V. G., Gurzadyan, G. G., & Nikogosyan, D. N. Handbook of nonlinear optical crystals. *Springer* (2013).
