# Supplementary material for: Frequency conversion of highly charged femtosecond spatiotemporal optical vortices in cascaded nonlinear crystals
Source: Nat Commun. 2026 Jul 29;17:9180. doi: 10.1038/s41467-026-75464-0 (PMC13521892; doi:10.1038/s41467-026-75464-0)
Supplement: Supplementary file 2 [file 41467_2026_75464_MOESM2_ESM.pdf]

nature portfolio

Peer Review File

Frequency conversion of highly charged femtosecond spatiotemporal optical vortices in cascaded nonlinear crystals

Corresponding Author: Professor Yi Liu

This file contains all reviewer reports in order by version, followed by all author rebuttals in order by version.

Version 0:

Reviewer comments:

Reviewer #1

(Remarks to the Author)
The manuscript investigates a cascaded sum frequency generation (SFG) process to produce third harmonic light, combining it with spatiotemporal optical vortices to achieve very high topological charge (up to $\ell = 40$). Although the results are interesting and merit publication, in my view the work lacks the novelty and impact required for Nature Communications. Similar approaches have already been reported in Refs. [21, 22]; the principal difference here appears to be the use of two crystals in sequence implementing the same SFG process—effectively applying the same process twice in a cascade.
The text is generally well written and the ideas are clearly presented, but several points need the authors' attention:
1. The manuscript alternates between the terms "SFG" and "SHG" as though they refer to the same process. Please standardize the terminology and correct any instances where "SHG" is used in place of "SFG" (or vice versa).
2. Line 180: It is not clear how the value of 299 fs is obtained ("…299 fs temporal delay after propagation through the 1 mm thick crystal…"). Please explain the reasoning and show the intermediate steps or provide a reference so that readers can reproduce this result.
3. Please clarify how the wavelength of 266 nm shown, for example, in Figs. 5 and 6 is obtained. If the inputs are 800 nm and 400 nm in an SFG scheme, the manuscript should explicitly state the calculation and assumptions that lead to the stated output wavelength and guide the reader through the reasoning.

Reviewer #2

(Remarks to the Author)
In this work the authors presents experimental work for Second-Harmonic-Generation and Sum-Frequency-Generation of a Spatiotemporal Optical Vortex (STOV) field. STOVs are relatively a new subject of study. They are a natural extension of the very well-known optical vortex from the spatial domain to the spatio-temporal domain where a spatial singularity in the former case is extended to a singularity in space-time (or equivalently in space-frequency). STOV was also demonstrated experimentally in nonlinear optics, both in the perturbative regime (e.g. SHG which the authors mention) and recently, in the non-perturbative regime – for High-Harmonic-Generation (see https://doi.org/10.1038/s41566-025-01699-w). As this is a very recent publication I assume the authors had no opportunity to mention it yet(and I recommend that they do so).
In the current study STOV is studied in perturbative nonlinear optics for cascaded processes, and as a result the output field can possess high values of the topological charge characterizing the singularity of STOVs. It is the first time, to the best of my knowledge, that STOVs are demonstrated for cascaded processes.
The manuscript is written exceptionally well – very clear and comprehensive while elaborating on important issues such as phase-matching considerations and spatio-temporal walk-off.
In my opinion, the work is suitable for publication in Nature Communications.

Reviewer #3

(Remarks to the Author)
This manuscript presents a valuable experimental study on cascaded second harmonic generation (SHG) and sum frequency generation (SFG) of femtosecond spatiotemporal optical vortices (STOVs) with high topological charges (TC), achieving TC values up to 40 and wavelength tunability across the UV-visible spectrum. The work provides a practical methodology for generating STOVs with large TCs and diverse wavelengths, and the experimental design is rigorous with compelling results, making it a meaningful contribution to the nonlinear optics and photonics communities. Therefore, I do recommend this manuscript for publication in Nature Communication after addressing the points listed below.

1.BBO crystals have been employed for wavelength tuning, SHG, and SFG processes, but critical phase-matching bandwidths (or tolerated wavelength ranges) are not provided. This information is essential to evaluate the wavelength tuning and SHG conversion efficiency, so it is recommended to supplement these parameters.
2.The manuscript notes that the type I SHG BBO produces orthogonally polarized fundamental and second harmonic fields, and the authors adjusted the crystal's azimuthal angle to generate parallel polarization components for SFG. It would be helpful to explain the rationale for not adopting type II phase matching, as it inherently accommodates orthogonal polarizations in certain configurations--which could potentially align with the natural polarization output of the type I SHG process without the need for azimuthal angle adjustment and associated SHG efficiency loss.
3.The study uses critical phase matching (CPM) for BBO crystals, but the potential impact of spatial walk-off on the harmonic STOV mode quality--especially for thicker crystals (~0.5 mm and 1 mm)--is not addressed. It is recommended to clarify its contribution to the observed mode degradation.
4.Diffraction patterns provide indirect evidence of topological charge but may mask subtle mode distortions caused by spatial walk-off, astigmatism, or beam separation. It is recommended to measure and present the field profiles (e.g., 2D intensity maps), which would allow for a quantitative assessment of whether the harmonic modes retain the desired spatio temporal structure or suffer from degradation due to crystal-induced effects.

Reviewer #4

(Remarks to the Author)
The submitted manuscript described highly charged femtosecond STOV lights were generated by two separated second-order nonlinear optical processes. The manuscript was well prepared with data in detail. Here are some questions and suggestions.
1. Usually, cascaded frequency conversion refers to several frequency conversion processes are happened in the same single nonlinear medium. I am not sure if "cascaded frequency conversion" is a good description here.
2. In the paper, cascaded frequency conversion is two separated second-order nonlinear optical processes. And the conservation of the spatiotemporal topological charge during second-order nonlinear process had already been experimentally proved in 2021 [ref 21] in the SHG process. Then, there is no new important discover except for highly TC. The novelty and importance should be shown clearly.
3. Since highly TC is the key point of the paper, it should be studied in detail. The largest TC in the paper is 60. Is there any limitation for the largest TC? What kinds of parameters affect the achievement of highly TC in the paper? How about the basic parameters, for example the energy conversion efficiency, during the SHG or SFG process with different high TC?
4. In Fig.4b, why the central part is much stronger than the sideband, which is different from other results with lower TC?

Version 1:

Reviewer comments:

Reviewer #1

(Remarks to the Author)
The authors have improved the manuscript by drawing attention to its novelty, mainly characterized by the extension of the parameter space for STOV generation and by pushing the generated wavelengths further into the ultraviolet region. They have also added new text to clarify the presented results.

Reviewer #2

(Remarks to the Author)
I read the reviewers concerns and the reply of the authors. It was my initial recommendation to publish this work in Nature Communications, and it is still my recommendation.

Reviewer #3

(Remarks to the Author)
The authors have addressed all concerns and revised the manuscript carefully. The manuscript can be accepted now.

Reviewer #4

(Remarks to the Author)

I think the authors have answered well all the questions. The revised paper is better than previous one. I think it can be published Nat. Communications right now. Only one question, from Fig.R3, the energy conversion decreased a lot for large TC, the application of it is a problem. Of course, for basic research it is OK.

Open Access This Peer Review File is licensed under a Creative Commons Attribution 4.0 International License, which permits use, sharing, adaptation, distribution and reproduction in any medium or format, as long as you give appropriate credit to the original author(s) and the source, provide a link to the Creative Commons license, and indicate if changes were made.
In cases where reviewers are anonymous, credit should be given to 'Anonymous Referee' and the source.
The images or other third party material in this Peer Review File are included in the article's Creative Commons license, unless indicated otherwise in a credit line to the material. If material is not included in the article's Creative Commons license and your intended use is not permitted by statutory regulation or exceeds the permitted use, you will need to obtain permission directly from the copyright holder.
To view a copy of this license, visit https://creativecommons.org/licenses/by/4.0/

Replies to the reviewers

Reply to the Comments of Reviewer 1

The manuscript investigates a cascaded sum frequency generation (SFG) process to produce third harmonic light, combining it with spatiotemporal optical vortices to achieve very high topological charge (up to $\ell = 40$). Although the results are interesting and merit publication, in my view the work lacks the novelty and impact required for Nature Communications. Similar approaches have already been reported in Refs. [21, 22]; the principal difference here appears to be the use of two crystals in sequence implementing the same SFG process—effectively applying the same process twice in a cascade.

Response: We thank Reviewer 1 for his/her time and constructive feedback, and especially for acknowledging that our "results are interesting and merit publication." We have carefully addressed all the specific points raised, as detailed below. As to the concern of novelty and impact, we would like to respectfully clarify and highlight the significant advances our work provides beyond the foundational studies in Refs. [21, 22]:

(1) Exploring an Unprecedented Parameter Space. While Refs. [21,22] demonstrated the principle of topological charge conservation in SHG for ℓ=1, our work very significantly exceeds this value by more than an order of magnitude (e.g., up to ℓ=40) – not merely in theory but in actual practice.

(2) Pushing this fundamental interaction into the high-charge regime is a game changer from the point of view of both applications and angular momentum conservation in this system. In many systems in nature the phase winding to achieve high values of angular momentum is often confounded by dissipation mechanisms, such as environment perturbation, unperfect reflection, beam propagation, spatiotemporal astigmatism, etc. A forty-fold increase is therefore astonishing.

(3) Extending the Ultraviolet Frontier for STOVs. While previous STOV demonstrations have been confined to the visible or near-infrared, our cascaded SFG scheme generates STOVs at 266 nm — the first, to our knowledge, in the ultraviolet spectral range. This wavelength extension is a key outcome, significantly broadening the potential application space of STOVs (e.g., in ultrafast UV spectroscopy or photochemistry).

(4) Providing a Tunable Methodology from Principle to Practical. Beyond a proof-of-principle demonstration, we provide a systematic study on wavelength tunability of harmonic STOVs and the practical limits imposed by crystal thickness. This transforms the principle into a versatile methodology for generating high-charge, wavelength-agile STOVs across the visible and UV spectrum.

In view of this concern of the reviewer, we have revised the abstract and introduction section to more clearly frame our work within this context, highlighting these points of novel contribution. We believe that together, these aspects--high charge, UV generation, and tunability study--constitute a significant step beyond prior art and offer substantial impact to the fields of nonlinear optics and structured light.

The text is generally well written and the ideas are clearly presented, but several points need the authors' attention:

Comment 1: *The manuscript alternates between the terms "SFG" and "SHG" as though they refer to the same process. Please standardize the terminology and correct any instances where "SHG" is used in place of "SFG" (or vice versa).*

Response: We thank the reviewer for this correction. We have thoroughly reviewed the manuscript and standardized the terminology. "SHG" now refers exclusively to second-harmonic generation (800 nm → 400 nm), and "SFG" to the sum-frequency generation process (800 nm + 400 nm → 266 nm).

Comment 2: *Line 180: It is not clear how the value of 299 fs is obtained ("…299 fs temporal delay after propagation through the 1 mm thick crystal…"). Please explain the reasoning and show the intermediate steps or provide a reference so that readers can reproduce this result.*

Response: We thank the reviewer for requesting clarification. The value is derived from the group velocity mismatch (GVM) in the BBO crystal. For Type-I phase-matching, the group velocities at 800 nm and 400 nm are different. Based on the dispersion properties of BBO [Dmitriev, V. G., Gurzadyan, G. G., & Nikogosyan, D. N. (2013). *Handbook of nonlinear optical crystals*. Springer], the group velocity mismatch is approximately $\Delta(1/v_g) \approx 194$ fs/mm. Therefore, for a 1 mm thick crystal, the temporal walk-off is $\Delta t = L \times \Delta(1/v_g) \approx 194$ fs. The figure of 299 fs was a typographical error in our initial submission. We have corrected this to "~194 fs" in the revised text (last paragraph of page 6 and corresponding Supplementary text) and added a reference for clarity.

Comment 3: *Please clarify how the wavelength of 266 nm shown, for example, in Figs. 5 and 6 is obtained. If the inputs are 800 nm and 400 nm in an SFG scheme, the manuscript should explicitly state the calculation and assumptions that lead to the stated output wavelength and guide the reader through the reasoning.*

Response: The output wavelength (λ_3) in an SFG process is given by $1/\lambda_3 = 1/\lambda_1 + 1/\lambda_2$, where λ_1 and λ_2 are the input wavelengths. Substituting $\lambda_1 = 800$ nm and $\lambda_2 = 400$ nm yields: $1/\lambda_3 = 1/800 + 1/400 = 0.00375$ nm^{-1}, therefore $\lambda_3 \approx 266.7$ nm. We have added this calculation at the first mention of the third-harmonic generation in the Results section for clarity (page 5, third paragraph).

In all, we thank the reviewer for the valuable questions and suggestions. We hope that revisions in the manuscript and the above replies address the concerns and improve the manuscript.

Reply to the Comments of Reviewer 2

In this work the authors presents experimental work for Second-Harmonic-Generation and Sum-Frequency-Generation of a Spatiotemporal Optical Vortex (STOV) field. STOVs are relatively a new subject of study. They are a natural extension of the very well-known optical vortex from the spatial domain to the spatio-temporal domain where a spatial singularity in the former case is extended to a singularity in space-time (or equivalently in space-frequency). STOV was also demonstrated experimentally in nonlinear optics, both in the perturbative regime (e.g. SHG which the authors mention) and recently, in the non-perturbative regime – for High-Harmonic-Generation (see https://doi.org/10.1038/s41566-025-01699-w). As this is a very recent publication I assume the authors had no opportunity to mention it yet(and I recommend that they do so).

In the current study STOV is studied in perturbative nonlinear optics for cascaded processes, and as a result the output field can possess high values of the topological charge characterizing the singularity of STOVs. It is the first time, to the best of my knowledge, that STOVs are demonstrated for cascaded processes.

The manuscript is written exceptionally well – very clear and comprehensive while elaborating on important issues such as phase-matching considerations and spatio-temporal walk-off.

In my opinion, the work is suitable for publication in Nature Communications.

Response: We are very grateful to Reviewer 2 for your strong endorsement of our work and your positive assessment of its suitability for Nature Communications. We also thank the reviewer for bringing the very recent and highly relevant work on STOVs in high-harmonic generation (HHG) to our attention. We agree this is an exciting development that further underscores the broad potential of STOVs in nonlinear optics. We have now cited this important reference in the revised Discussion section (following the sentence on HHG potential). It provides strong supporting evidence that our methodology — capable of generating high-charge STOV driver fields — is well-suited for advancing such extreme nonlinear experiments.

Reply to the Comments of Reviewer 3

This manuscript presents a valuable experimental study on cascaded second harmonic generation (SHG) and sum frequency generation (SFG) of femtosecond spatiotemporal optical vortices (STOVs) with high topological charges (TC), achieving TC values up to 40 and wavelength tunability across the UV-visible spectrum. The work provides a practical methodology for generating STOVs with large TCs and diverse wavelengths, and the experimental design is rigorous with compelling results, making it a meaningful contribution to the nonlinear optics and photonics communities. Therefore, I do recommend this manuscript for publication in Nature Communication after addressing the points listed below.

Response: We sincerely thank Reviewer 3 for his/her positive assessment of our work as a "valuable experimental study" and "meaningful contribution," and for the recommendation for publication in Nature Communications. The detailed and constructive comments have significantly helped us improve the manuscript. We address each point below.

Comment 1: *BBO crystals have been employed for wavelength tuning, SHG, and SFG processes, but critical phase-matching bandwidths (or tolerated wavelength ranges) are not provided. This information is essential to evaluate the wavelength tuning and SHG conversion efficiency, so it is recommended to supplement these parameters.*

Response: We thank the reviewer for raising this important point. The phase-matching bandwidth is indeed crucial for evaluating wavelength tunability and conversion efficiency.

For a 100-μm-thick BBO crystal, the acceptance bandwidth (FWHM) can be estimated from the crystal's dispersion. Our calculations yield the following values for the specific processes and phase-matching angles used in our experiment:

- SHG process (800 nm → 400 nm, Type-I, θ = 29.2°): The phase-matching bandwidth is approximately $\Delta\lambda \approx 49$ nm for the fundamental wavelength.
- SFG process (800 nm + 400 nm → 266 nm, Type-I, θ = 43.0°): The acceptance bandwidths are approximately $\Delta\lambda \approx 12.5$ nm for the 800 nm beam and $\Delta\lambda \approx 5.5$ nm for the 400 nm beam, in both cases with a fixed wavelength of the other beam for SFG.

These calculated bandwidths confirm that the spectral tunability we demonstrated for the second harmonic (~16.5 nm around 400 nm, as shown in Supplementary Fig. 5) is well-supported within the phase-matching limits. In the wavelength-tuning experiment, by shifting the spectral position of the phase singularity, we selectively coupled different spectral components into the phase-matched region, enabling the observed tuning range. we have conducted additional experiments to demonstrate wavelength tuning for the third harmonic (266 nm, see Fig.R1). These new results, which confirm the extended tunability of our sequential frequency conversion scheme, are now presented in the newly added Supplementary Fig. 5 of the revised manuscript.

We have referenced these key parameters and their implications in the revised Results section (Page 5, 2nd Paragraph; Page 6, 1st Paragraph) of the main text, with full derivations provided in the Supplementary Information (Section S[4]).

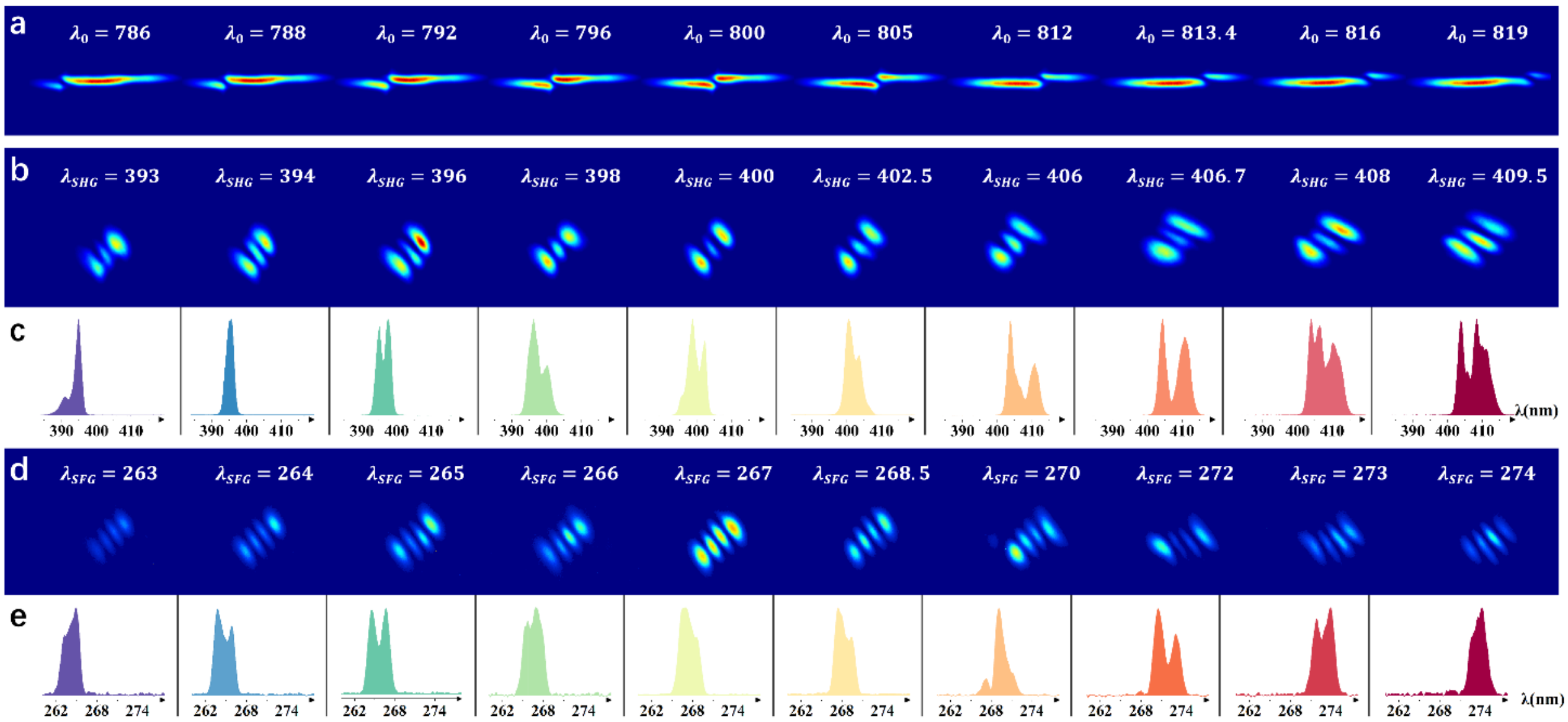

Fig. R1. Tunability of the second and third harmonic STOV field.

Comment 2: *The manuscript notes that the type I SHG BBO produces orthogonally polarized fundamental and second harmonic fields, and the authors adjusted the crystal's azimuthal angle to generate parallel polarization components for SFG. It would be helpful to explain the rationale for not adopting type II phase matching, as it inherently accommodates orthogonal polarizations in certain configurations--which could potentially align with the natural polarization output of the type I SHG process without the need for azimuthal angle adjustment and associated SHG efficiency loss.*

Response: We thank the reviewer for this insightful suggestion. Our choice of type I BBO was primarily based on its availability in our laboratory at the time of the experiments. Type I crystals are also a common commercial option for sum-frequency generation, and we found that this configuration fully met our experimental requirements.

That said, we acknowledge that a direct comparison between type I and type II would be informative, and we plan to explore this in future work. We sincerely appreciate the reviewer's suggestion for guiding this direction.

Comment 3: *The study uses critical phase matching (CPM) for BBO crystals, but the potential impact of spatial walk-off on the harmonic STOV mode quality--especially for thicker crystals (~0.5 mm and 1 mm)--is not addressed. It is recommended to clarify its contribution to the observed mode degradation.*

Response: The reviewer raised an important question. We believe that the spatial walk-off effect and the group velocity dispersion (GVM) of the SHG crystal are both important for the STOV mode quality, especially for the third harmonic STOV in the sum-frequency crystal.

For the spatial walk-off effect, it is known that the walk-off angle of the type I BBO crystal for 800 nm/ 400nm conversion is about $\rho \approx 4.28^o$[Dmitriev, V. G., Gurzadyan, G. G., & Nikogosyan, D. N. (2013). *Handbook of nonlinear optical crystals*. Springer]. This leads to a spatial separation of

$\Delta x = d \cdot tan(\rho)$ at the rear surface of the SHG crystal, corresponding to Δx ~ 7.5 μm and 75 μm for d = 0.1 and 1mm thick crystal respectively. This is not critical for the second harmonic generation of STOV, since we used a beam diameter of 4.0 mm. In contrast, this spatial walk-off effect matters for the third harmonics generation in the BBO crystal for SFG. The SFG and SHG crystals were in a cascaded configuration with typically 20 mm distance between them. The 4.28° walk-off angle of the 800 nm and 400 nm beams leads to a transverse separation Δx ~ 1.48 mm on the surface of the SFG crystal. This can lead to degradation in SFG process due to the substantial separation between the two beams. Here, one notice that the spatial separation of the 800nm/400nm beams is mainly due to the free propagation of the 800nm/400nm beams between the two crystals, not the propagation inside the SHG crystal. Therefore, the crystal thickness itself does not lead to substantial difference for the spatial walk-off effect.

For the group velocity dispersion effect, the thickness of the crystal plays an important role. The group delay dispersion between the 800 nm and 400 nm pulses is about 19.4 fs for 100 μm thick type I BBO crystal. In our experiment, the input laser pulse for the 4f pulse shaper exhibits a duration of 35 fs. After the pulse shaping into a STOV field, the 800 nm pulse duration broadens to tens or hundreds of femtoseconds, depending on the topological charge ℓ (see Fig. R2). Consequently, the 194 fs group delay between the 800 nm/400nm for 1 mm thick SHG crystal will be detrimental for the sum frequency generation inside the second BBO crystal. For this GVD effect, the free propagation in air has a minor contribution due to the weak dispersion of air comparing to BBO crystal.

Taking into consideration both the spatial walk-off effect and the GVM, we conclude that the thicker BBO crystal (0.5 mm and 1 mm) lead to a stronger GVM effect than the thin one. Therefore, the mode degradation of the 266 nm STOV for thicker BBO was mainly attributed to the temporal effect, rather than the spatial walk-off effect, since the latter is almost the same for SHG of different crystals.

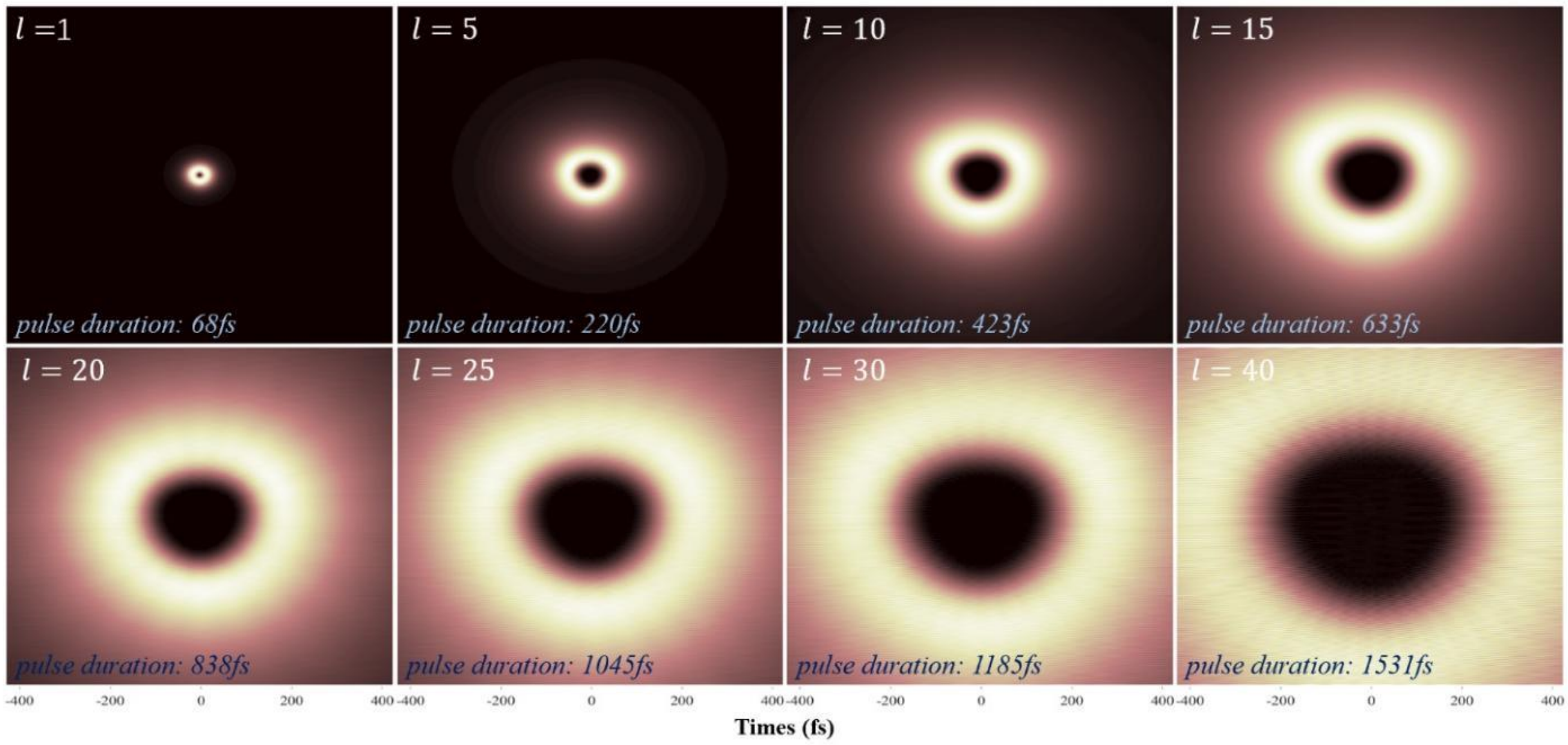

Fig.R2. Simulated dependence of STOV pulse duration on topological charges.

Comment 4: *Diffraction patterns provide indirect evidence of topological charge but may mask subtle mode distortions caused by spatial walk-off, astigmatism, or beam separation. It is recommended to measure and present the field profiles (e.g., 2D intensity maps), which would allow for a quantitative assessment of whether the harmonic modes retain the desired spatio-temporal structure or suffer from degradation due to crystal-induced effects*

Response: The reviewer is right that the diffraction method provides indirect evidence and the subtle distortion of the STOV may be masked. It will be ideal to perform 3D reconstruction of the STOV wave packet with methods such as time-resolved interference [15, 21]. In view of our generated STOV pulses with pulse duration of tens to hundreds of femtoseconds, a few-cycle pulse at the 400 nm or 266 nm wavelength will be necessary for this interference method. This will be technically demanding and challenging, beyond the scope of the current study. In the future study, it will be our next step to construct the spatiotemporal profile of the STOV at 400 nm and 266 nm with some other newly emerging methods such as single-frame spatially resolved spectral interferometry [ACS Photonics, 9, 2802 (2022)] or spatiotemporal wavefront sensor [Optica, 12, 1884 (2025)].

Finally, we would like to thank the reviewer for the valuable comments and concerns. We hope that the above response and the revisions of the manuscript address these concerns and improve the manuscript.

Reply to the Comments of Reviewer 4

Comment 1: *Usually, cascaded frequency conversion refers to several frequency conversion processes are happened in the same single nonlinear medium. I am not sure if "cascaded frequency conversion" is a good description he.*

Response: We thank the reviewer for this precise semantic point. We agree with the reviewer that "cascaded frequency conversion" usually refers to processes in the same nonlinear medium. To avoid confusion with the established meaning of "cascaded nonlinearity" we have changed the title and terminology throughout the manuscript. The revised title is "Frequency conversion of highly charged femtosecond spatiotemporal optical vortices in cascaded nonlinear crystals". We noticed that the terminology "cascaded nonlinear crystals" for efficient SHG and SFG has been widely used in the literatures, for example:

- Highly efficient single-pass sum frequency generation by cascaded nonlinear crystals. Opt. Lett. 40, 5526(2015).
- Concept for power scaling second harmonic generation using a cascade of nonlinear crystals. Opt. Express 23, 15921(2015).
- Efficient third-harmonic generation of broadband picosecond radiation by employing spectrally noncritical phase-matching KDP crystal and cascaded LBO crystals. Opt. Laser Technol. 141, 107105(2021).

Comment 2: *In the paper, cascaded frequency conversion is two separated second-order nonlinear optical processes. And the conservation of the spatiotemporal topological charge during second-order nonlinear process had already been experimentally proved in 2021 [ref 21] in the SHG process. Then, there is no new important discover except for highly TC. The novelty and importance should be shown clearly.*

Response: We appreciate the reviewer's concern and suggestion that the novelty and importance of our work should be further improved. In our opinion, although the conservation of topological charge in a single second-harmonic generation (SHG) process in the simplest case ($l = 1$) has been previously demonstrated, our work represents a substantial advance beyond this established fact through four keys.

(1) Exploring an Unprecedented Parameter Space. While Refs. [21,22] demonstrated the principle of topological charge conservation in SHG for ℓ=1, our work very significantly exceeds this value by more than an order of magnitude (e.g., up to ℓ=40) – not merely in theory but in actual practice.

(2) Pushing this fundamental interaction into the high-charge regime is a game changer from the point of view of both applications and angular momentum conservation in this system. In many systems in nature the phase winding to achieve high values of momentum is often confounded by dissipation mechanisms, such as environment perturbation, unperfect reflection, beam propagation, spatiotemporal astigmatism, etc. A forty-fold increase is therefore astonishing.

(3) Extending the Ultraviolet Frontier for STOVs. While previous STOV demonstrations have been confined to the visible or near-infrared, our cascaded SFG scheme generates STOVs at 266 nm — the first, to our knowledge, in the ultraviolet spectral range. This wavelength extension is a

key outcome, significantly broadening the potential application space of STOVs (e.g., in ultrafast UV spectroscopy or photochemistry).

(4) Providing a Tunable Methodology from Principle to Practical. Beyond a proof-of-principle demonstration, we provide a systematic study on wavelength tunability of harmonic STOVs and the practical limits imposed by crystal thickness. This transforms the principle into a versatile methodology for generating high-charge, wavelength-agile STOVs across the visible and UV spectrum.

In summary, the novelty of our work lies in several advances: pushing STOVs into the high-TC regime and, within this new regime, establishing the additivity law for conversion in a cascaded configuration while expanding operational wavelengths to the UV. This constitutes a systematic exploration that opens new directions, distinguishing it fundamentally from prior verification of the basic conservation law in a low-TC, single-step second harmonics generation process.

In view of this concern of the reviewer, we have revised the abstract and introduction section to more clearly frame our work within this context, highlighting these points of novel contribution.

Comment 3: *Since highly TC is the key point of the paper, it should be studied in detail. The largest TC in the paper is 60. Is there any limitation for the largest TC? What kinds of parameters affect the achievement of highly TC in the paper? How about the basic parameters, for example the energy conversion efficiency, during the SHG or SFG process with different high TC?*

Response: These are excellent questions that allow us to deepen the discussion on the limits and parameters governing high topological charge generation. In addition, we have performed new experiments as to the conversion efficiency during the SHG and SFG for larger topological charge. In the following, we would like to respond to your comments in their order.

- *Is there any limitation for the largest TC? What kinds of parameters affect the achievement of highly TC in the paper?*

The largest topological charge for the fundamental wavelength, the second harmonic and the third harmonic are limited by different factors. For the fundamental pulse, the spatial resolution of the spatial light modulator (SLM) imposes the most important constrain. On the SLM, a spiral phase distribution was loaded for the generation of 800 nm STOV. Due to the finite pixel size (Δ = 12 μm in our case) of the SLM, the maximum TC is limited due to that a complete phase winding of $l \times 2\pi$ cannot be implemented for the area around the phase singularity on the SLM for large topological charge l. This can be estimated in the following. Our 800 nm incident beam has a radius of $r_0 = 2.0$ mm. Let us take the criterion that an inner area with radius of r_c carrying 10% of the total beam energy is allowed to carry incomplete phase vortex while the outside beam takes the entire desired phase vortex of $l \times 2\pi$. In another word, the phase distribution inside the circle with radius r_c is "blurred", which accounts for 10% of the total beam energy. Considering a Gaussian beam with radius r_0, the 10% energy portion corresponds to a diameter of $r_c = 0.23 r_0$. Therefore, we assume that the circle with radius r_c carries the complete phase of $l_{max} \times 2\pi$. On the SLM, for implementation of a phase 2π, we further assume that 8 pixels are required, considering that at least 2 pixels are necessary for

the sampling of a π/2 phase variation. We then come to the estimation $\frac{2\pi r_c}{\Delta\times l_{max}} \geq 8$, which results in $l_{max} \leq \frac{2\pi r_c}{8\Delta}$. With the parameters of our experiments, we obtained the largest topologic charge to be $l_{max} = 30$. This is in good agreement with our experiment achievements of maximum topological of $l = 40$ for the 800 nm beam.

For the 400 nm STOV, the SHG crystal just imposes a limitation on the bandwidth of frequency conversion. This may lead to a reduced frequency bandwidth of the 400 nm pulse. However, the second harmonic generation process does not pose any constrain on the largest topological charge. This is in line with our observation that clear diffraction pattern of 400 nm STOV pulse can be obtained with large TC up to $l_{400} = 60$.

For the generation of the third harmonic STOV, the spatial walk-off and the group velocity dispersion of the 800 nm and 400 nm pulses both impose important constrain. As we replied to the 3rd comment of Reviewer 3, the mode quality of the third harmonic STOV is significantly reduced, comparing to that of the 800 nm and 400 nm. Therefore, the largest TC for the 266 nm pulse was found to be $l_{266} = 18$, much less than those of 800 nm and 400nm.

In summary, the largest topological charge of the 800 nm fundamental pulse is mainly limited by the spatial resolution (pixel size) of the SLM and incident beam size. For the second harmonic, the largest topological charge is not limited by the nonlinear conversion process. For the third harmonic generation with our cascaded configuration, the spatial walk-off and the GVD of the 800 nm and 400 nm pulses pose important constrain.

In view of this insightful comments of the reviewer, we have added corresponding discussion in the revised manuscript. The relevant contents appear in Section 1 of Supplementary Information.

- *How about the basic parameters, for example the energy conversion efficiency, during the SHG or SFG process with different high TC?*

The reviewer raised an important question here. As we have shown in the previous submitted manuscript, the conversion efficiency for both the SHG and SFG decrease for higher TC, as presented in the previous Fig. 5. In view of your concern as to higher TC, we have performed additional measurement during the manuscript revision. Now we extend the TC of both the 400 nm and 266 nm to even higher TC. The results are presented in Fig. R3 and in the newly added Fig. 5a (for SHG) and Fig. 5b (for SFG) in the revised manuscript. It is obvious that higher TC leads to a reduced conversion efficiency. The primary cause for this efficiency attenuation is the reduced energy flux density in high-TC STOV fields. As the TC increases, the STOV field experiences temporal pulse broadening and an increase in transverse beam size (Fig. R2), leading to a substantial drop in the fundamental intensity. This results in the observed sharp decline in harmonic conversion efficiency at high topological charges. In view of this comment, we have added discussion in the revised manuscript. The relevant contents appear in paragraph 4 of Page 4.

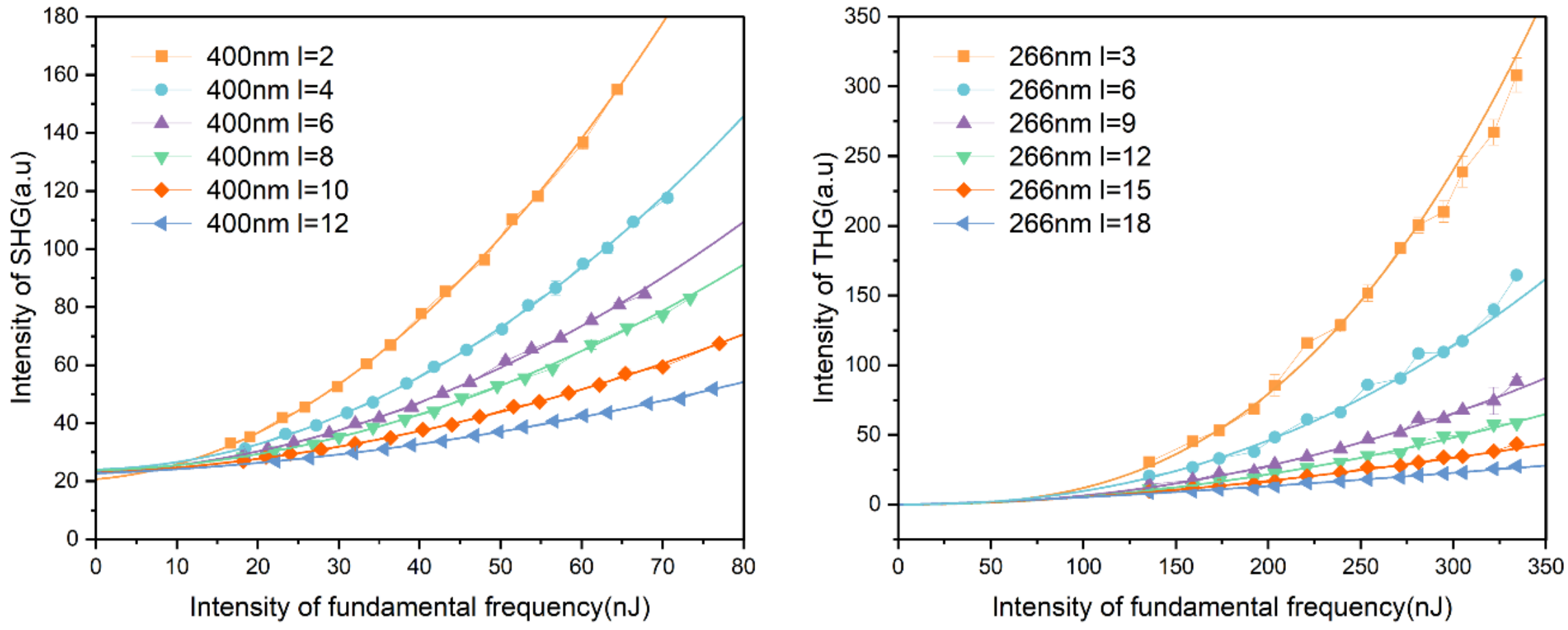

Fig. R3. The second and third harmonic signal with different topological charges for increasing incident energy of the 800 nm fundamental pulse.

Comment 4: *In Fig.4b, why the central part is much stronger than the sideband, which is different from other results with lower TC?*

Response: The reviewer raised an interesting question. In Fig. 4b, the second-harmonic diffraction pattern with the high topological charge $\ell(2\omega) = 60$ exhibits a much stronger central intensity compared to the sidebands. This is different from the results with lower topological charges where the intensity is more uniformly distributed between the center and sidebands.

We think this phenomenon mainly results from the spatial resolution (pixel size) of the SLM. As we have discussed in the response to the 3rd comment of Reviewer 3, on the SLM the center of the helical phase function is expected to be an ideal phase singularity in theory. However, as the topological charge increases, this singularity becomes susceptible to distortion. This distortion is related to the spatial resolution of the SLM. For a fixed pixel resolution, a higher topological charge leads to a larger distorted region around the center. Consequently, the light in this central region does not carry a well-defined helical phase but rather contributes to a noisy background. Therefore, we observed that the central region of diffraction pattern becomes stronger as the topological charge increases.

To confirm the above reasoning, we have recently performed a simplified simulation as to this effect. In Fig. R4, we presented the calculated diffraction pattern of the 400 nm STOV for $\ell\,(2\omega) = 60$. A central distorted region of the SLM is assumed by "zeroing" the phase in that area. We noticed that a bright central spot becomes significant for the case of phase "zeroing" in the center of the SLM, in agreement with the observation in Fig. 4. We therefore conclude that this central part origins mainly from the phase distortion around the phase singularity on the SLM due to its spatial resolution.

In view of this comment of the reviewer, we have added a short discussion in the revised manuscript. The relevant contents appear in paragraph 2 of Page 4.

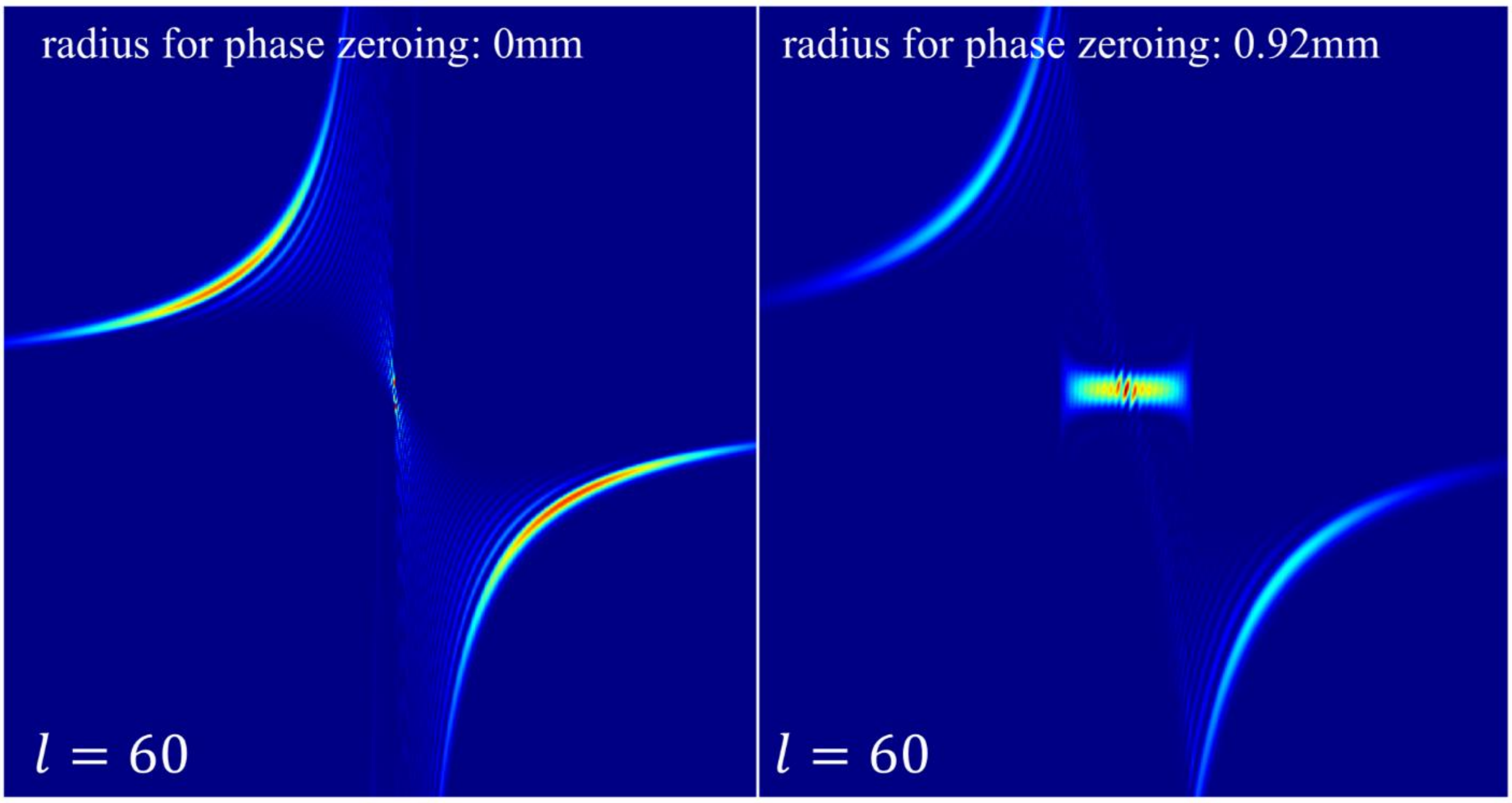

Fig. R4. (a) and (b), calculated diffraction pattern of the 400 nm STOV obtained without (with) a distorted central region around the phase singularity on the SLM.

In all, we sincerely thank the reviewer for the constructive questions and comments. We hope the above response and the revisions in the manuscript have addressed the concerns and comments of the reviewer and strengthened the manuscript.
